# Reticulophagy and Ribophagy: Regulated Degradation of Protein Production Factories

**DOI:** 10.1155/2012/182834

**Published:** 2012-02-28

**Authors:** Eduardo Cebollero, Fulvio Reggiori, Claudine Kraft

**Affiliations:** ^1^Department of Cell Biology and Institute of Biomembranes, University Medical Centre Utrecht, 3584 CX Utrecht, The Netherlands; ^2^Department of Biochemistry and Cell Biology and Institute of Biomembranes, Utrecht University, 3508 TD Utrecht, The Netherlands; ^3^Max F. Perutz Laboratories, University of Vienna, 1030 Vienna, Austria

## Abstract

During autophagy, cytosol, protein aggregates, and organelles are sequestered into double-membrane vesicles called autophagosomes and delivered to the lysosome/vacuole for breakdown and recycling of their basic components. In all eukaryotes this pathway is important for adaptation to stress conditions such as nutrient deprivation, as well as to regulate intracellular homeostasis by adjusting organelle number and clearing damaged structures. For a long time, starvation-induced autophagy has been viewed as a nonselective transport pathway; however, recent studies have revealed that autophagy is able to selectively engulf specific structures, ranging from proteins to entire organelles. In this paper, we discuss recent findings on the mechanisms and physiological implications of two selective types of autophagy: ribophagy, the specific degradation of ribosomes, and reticulophagy, the selective elimination of portions of the ER.

## 1. Introduction

Autophagy is a degradative process that allows cells to maintain their homeostasis in numerous physiological situations. It is required, for example, to face prolonged starvation periods and nutritional fluctuations in the environment, developmental tissue remodeling, organelle quality control, and immune responses [1, 2]. In addition, this pathway has been implicated in the physiopathology of multiple diseases [3, 4]. Autophagosomes are the hallmark of autophagy. These double-membrane vesicles are generated in the cytosol and during their formation they engulf the cargo to be delivered into the mammalian lysosomes or yeast and plant vacuoles for degradation [5]. Two types of autophagy have been described: selective and non-selective autophagy. During non-selective autophagy bulk cytosol, including organelles, is randomly sequestered into autophagosomes. On the other hand, during selective autophagy, a specific cargo is exclusively enwrapped by double-membrane vesicles, which contain little cytoplasm with their size corresponding to that of their cargo [6].

Autophagy progression relies on the function of the autophagy-related (Atg) proteins that mediate autophagosome biogenesis, selective cargo recognition, fusion with the lysosome/vacuole, or vesicle breakdown [5, 7, 8]. Upon nutritional stresses, fractions of the cytoplasm are consumed via autophagy and the resulting catabolic products are used as sources of energy or as building blocks for the synthesis of new macromolecules. In these situations autophagy is mainly considered as a non-selective process. Nonetheless an increasing number of selective types of autophagy are being described [6, 9] and these findings challenge the concept whether autophagosomes in fact sequester their cargo randomly.

## 2. Short Overview of Selective Types of Autophagy

One of the best-studied examples of selective autophagy is the biosynthetic cytoplasm to vacuole targeting (Cvt) pathway in the yeast *Saccharomyces cerevisiae*. During the Cvt pathway a protein oligomer composed of the vacuolar hydrolases aminopeptidase 1 (Ape1), *α*-mannosidase 1 (Ams1), and aspartyl aminopeptidase (Ape4), is delivered to the vacuolar lumen by small double-membrane vesicles [10–13]. Interestingly, this oligomer is also a specific cargo of autophagosomes under starvation conditions [14]. In higher eukaryotes autophagy also supports the selective destruction of intracellular pathogens, called xenophagy, and protein aggregates, named aggrephagy. In addition, metabolically dispensable or dysfunctional organelles can be specifically degraded by autophagy in both yeast and mammals. Examples of the latter include the exclusive elimination of superfluous or damaged mitochondria, termed mitophagy, and the selective consumption of excessive peroxisomes, called pexophagy [15, 16].

The underlying mechanisms of each of these pathways remain to be characterized in detail but some common principles are emerging. First, a receptor-like recognition of the cargo directing it to the autophagosome or alternatively recruiting the Atg machinery is required for all the selective types of autophagy. Second, the involvement of ubiquitin as a signaling molecule has been described for several selective types of autophagy in higher eukaryotes [17]. Several of the autophagosomal cargos can be degraded in a selective manner under specific conditions or in a random manner during bulk autophagy. It remains to be investigated in more detail how certain autophagy pathways can choose specific cargo in time and space. As the subject of selective autophagy pathways is covered in other reports in this special issue of the *International Journal of Cell Science*, in this review we will discuss the molecular principles and mechanisms underlying two selective types of autophagy that remain less well understood: ribophagy and reticulophagy.

## 3. Ribophagy: Mechanisms and Physiological Implications

**Figure 1:**
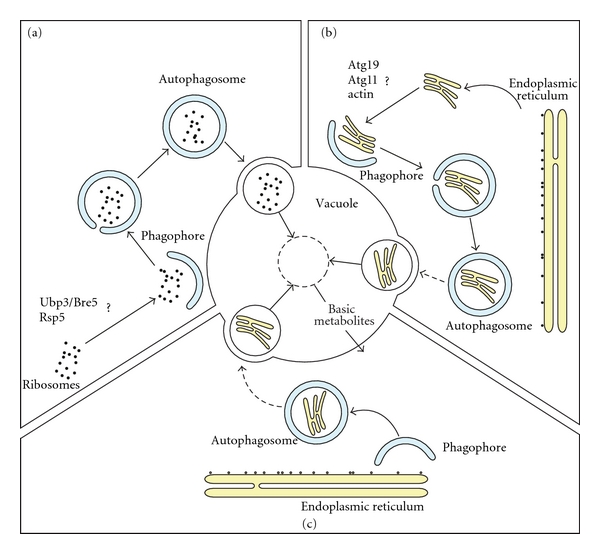
Mechanisms of ribophagy and reticulophagy in yeast. (a) A model for ribophagy. Under ribophagy inducing conditions, ribosomes are selectively engulfed into autophagosomes and subsequently degraded in the vacuole. The generated basic metabolites (amino acids, sugars, fatty acids etc.) are then recycled back to the cytoplasm for reuse or as a source of energy. ((b) and (c)) Models for reticulophagy. Under stress conditions, due to an accumulation of unfolded proteins and/or protein aggregates, a partial scission of the ER occurs and the formed fragments are specifically transported to the sites where autophagosome biogenesis takes place (b). ER stress triggers the recruitment of the Atg proteins onto or close to this organelle. There, possibly by utilizing the ER membranes, the Atg proteins mediate the formation of autophagosomes, which expand around the ER sections that have to be removed (c). The dashed arrows indicate that under specific ER stress conditions, autophagosomes do not fuse with the vacuole. Question marks highlight proteins that have been implicated in the transport and selection of the cargo in which the mechanism of action remains to be elucidated.

Since the discovery of autophagy, ribosomes have been detected in the interior of autophagosomes by electron microscopy [18, 19]. For a long time these large multiprotein complexes were viewed as a marker for bulk degradation of cytoplasm. However, it has recently been shown that ribosomes are turned over through a selective type of autophagy [20]. Accurate examination of ribosome fate under nutrient starvation conditions in yeast *S. cerevisiae* has revealed that these structures are more rapidly degraded compared to other cytoplasmic components, supporting the notion of a selective degradation process [20]. The involvement of autophagy in this event was demonstrated by uncovering that the transport of ribosomes to the vacuole relies on core autophagy components such as Atg1 and Atg7. A genetic screen in yeast designed to isolate mutant strains with a defect in ribosome turnover revealed that the ubiquitin protease Ubp3 and its cofactor Bre5 are required for this selective type of autophagy, however, not for bulk autophagy [20]. Importantly, a catalytically inactive mutant of Ubp3 also displayed a defect in the autophagy-mediated degradation of ribosomes indicating that ubiquitination plays a key role in this process. This selective autophagic turnover of ribosomes is now termed ribophagy [20] (Figure 1(a)).

## 4. Ribophagy and Ubiquitination

It remains to be investigated whether ubiquitination is important for either the regulation of signaling pathways triggering ribophagy or in dictating the specificity in the cargo selection. This latter possibility is evoked by the fact that ubiquitin-based modifications are a common theme in the selective elimination of specific structures in higher eukaryotes [17]. As Ubp3 interacts with and influences the ubiquitination status of Atg19 [21], a receptor protein of the Cvt pathway [22], it is plausible that Ubp3 could contribute to other selective types of autophagy in a similar manner. Further evidence for the involvement of ubiquitination in ribophagy comes from the finding that a decrease of the cytoplasmic levels of the ubiquitin ligase Rsp5 together with the deletion of *UBP3* results in a defect in the turnover of ribosomes higher than in the *ubp3*Δ cells [23]. Importantly, cytoplasmic proteins are normally degraded by autophagy in this strain. These findings imply that both ubiquitination and deubiquitination are crucial for the regulation of ribophagy. A reciprocal control mechanism has also been found to be important for the specific removal of midbody rings by autophagy during cytokinesis [24]. To understand the regulation and mechanism of ribophagy, it will be important to identify the targets of Ubp3/Bre5 and Rsp5 during this process.

## 5. Putative Physiological Roles of Ribophagy

What could be the physiological role of ribophagy? The deletion of *UBP3 *results in the inhibition of starvation-induced ribophagy and leads to cell death, without affecting general bulk autophagy [20]. These findings support the notion that not only bulk autophagy, but also ribosomal turnover is important for cell survival during nutrient limiting conditions. This does not come as a surprise as ribosomes constitute half of the cell's protein mass [25], and consequently, represent a major source of amino acids during times of nutrient deprivation. In addition, or alternatively, the importance of ribosomal degradation during starvation might be its contribution to the rapid and simultaneous downregulation of protein translation, a process that consumes large amounts of energy and amino acids.

Interestingly, a ribophagy-like process has also been proposed in plants. The endoribonuclease Rns2, a conserved member of the RNAse T2 protein family, is required for ribosomal RNA decay in plants [26]. Mutant cells lacking Rns2 activity fail to degrade ribosomal RNA. If this results in a failure of disassembling and/or degrading entire ribosomes has not yet been determined. Nevertheless, the defect in the turnover of ribosomal RNA suggests that Rns2 is a component of a ribophagy-like process in plants. The plant *rns2* mutants also exhibit this phenotype in nutrient rich conditions. This suggests that ribophagy might also serve a housekeeping function by recycling some of the ribosomal components such as amino acids and nucleotides. To date, only the degradation of ribosomal RNA has been studied. Consequently, the fate of ribosomal proteins as well as the existence of ribophagy in plants will require more detailed investigations.

## 6. Ribophagy in Higher Eukaryotes

Ribosomes have also been observed in the interior of autophagosomes in mammalian cells [18]. In particular, the relative abundance of proteins in MCF7 cells during amino acid starvation has been measured using quantitative mass spectrometry [27]. This approach has revealed that in mammalian cells ribosome degradation by autophagy occurs with different kinetics than that of other cytoplasmic proteins and organelles [27]. It has yet to be explored, however, whether a selective type of autophagy is responsible for the different turnover rates of ribosomes and cytoplasmic proteins. Additional evidence for the possible existence of ribophagy in higher eukaryotes comes from a murine study on neurodegeneration in Purkinje cells, where the disassembly of actively translating polysomes to nontranslational monosomes was observed among other changes [28]. Interestingly, a fraction of the free monosomes was specifically sequestered into autophagosomes suggesting that an autophagy-related pathway is involved in the selective degradation of ribosomes in these cells [28]. Thus, these neuronal cells appear to be an optimal model to study ribophagy and possibly gain additional insight into the involvement of both ubiquitination and the mammalian Ubp3 homologue Usp10 in the turnover of ribosomes in higher eukaryotes.

Autophagy of ribosomal proteins has also been demonstrated to serve an antimicrobial function. Several bacteria are directly captured in the cytosol by the autophagy adapter p62 or NDP52, and subsequently sequestered into autophagosomes to be delivered and degraded in lysosomes [29]. In the case of *Mycobacterium tuberculosis*, autophagy can also be used for its removal from the cell, however, through a different mechanism. Mycobacteria are phagocytosed by macrophages whereupon they delay phagosome maturation, thereby preventing their destruction in the lysosome. In the phagosomes, they persist and replicate often leading to lethal infections. Recently it has been shown that upon autophagy induction the cytosolic ribosomal protein rpS30 precursor FAU and ubiquitin are sequestered into autophagosomes in a p62-dependent manner [30]. In the mature autophagosome, these proteins are processed into peptides possessing antimicrobial properties that direct the killing of *Mycobacterium *[30]. Because of the involvement of p62, this antimicrobial turnover of ribosomal protein precursors appears to have all the characteristics of a selective type of autophagy.

An alternative role for ribophagy in cell homeostasis arises from the possibility that this pathway could also target defective ribosomes under normal growth conditions. In this scenario, by specifically eliminating nonfunctional, incorrectly assembled, and/or damaged ribosomes, ribophagy would have a quality control function. Avoiding the translation of incorrect and potentially harmful proteins might be crucial for cell homeostasis. Along this line, it is important to note that several diseases have been associated with specific mutations in ribosomal subunits [31]. The identification of such a quality control function, as well as the mechanism underlying it will be important directions for future analyses.

## 7. Protein Folding and ER Stress

While ribosomes located in the cytosol mainly translate cytoplasmic proteins, the synthesis of proteins that are secreted or reside in one of the organelles of the endomembrane system is mediated by ribosomes associated with the ER. As these newly synthesized proteins are cotranslationally translocated into the ER, a conspicuous amount of these molecules remains localized to this compartment. In order to prevent the accumulation of misfolded polypeptides, the ER counts on a specialized group of proteins, the so-called chaperones, which assist the folding of the nascent polypeptides or recognize misfolded proteins and mediate their refolding [32]. Under certain circumstances, this quality control function of the ER can be overcome by the natural occurrence of mutations or peculiar environmental conditions that affect general protein folding. This scenario can also be mimicked by expression of specific mutant proteins or treatment with particular chemical agents [33–37]. These situations may result in the accumulation of unfolded proteins and aggregates in the ER. Two interconnected safeguard mechanisms, the unfolded protein response (UPR) and the ER-associated degradation (ERAD), are in place to cope with misfolded protein buildups [38–40]. The UPR is an intracellular signaling cascade triggered by ER stress. This signal is transduced into cytoplasmic and nuclear actions aimed at increasing the inherent folding capacity of the ER and eliminating the misfolded proteins accumulated in this organelle. Among the responses initiated by the UPR are inhibition of general translation and upregulation of genes encoding ER chaperones and components of the ERAD machinery. The ERAD in turn, recognizes misfolded proteins and retrotranslocates these proteins into the cytoplasm where they are degraded by the ubiquitin-proteasome system. This elimination is mediated by the retrotranslocon complex, a multiprotein system seated in the ER membrane that facilitates the transport of unfolded proteins across the ER, catalyzes the polyubiquitination of the exported proteins, and mediates their delivery to the proteasome. Autophagy might serve a third cellular mechanism complementing the UPR and ERAD systems in coping with the harmful accumulation of unfolded or aberrant proteins in the ER.

## 8. Autophagy in ER Stress

Molecular events occurring upon autophagy induction are the association of Atg8/LC3 with autophagosomal membranes through its conjugation to the lipid phosphatidylethanolamine (PE) [41, 42] and the formation of autophagosomes. Yeast and mammalian cells subjected to different ER stresses exhibit levels of lipidated Atg8/LC3 similar to those displayed by starved cells [33, 37, 43, 44]. Additionally, light microscopy studies have revealed that ER stress induces the formation of autophagosomes in all eukaryotes analyzed [33, 44, 45]. Both this lipidation event and the formation of autophagosomes during ER stress can be blocked by chemical inhibitors of autophagy or Atg protein depletion [33, 37, 43, 44]. This, in combination with ultrastructural analyses of both yeast and mammalian cells following ER stress, which showed the induction of autophagosomes and autophagolysosomes, confirms the induction of an autophagy response upon ER stress [36, 43, 45, 46]. A detailed scrutiny of the luminal contents of these carriers has revealed that autophagosomes enclose portions of the ER [45, 46]. The amount of ER sequestered in their interior, however, depends on the nature and strength of the stimuli triggering the reticulophagy response. For example, when yeast are treated with the reducing agent dithiothreitol (DTT), which inhibits disulfide bond formation and thus prevents correct protein folding, autophagosomes are mostly filled with tightly stacked ER membrane cisternae [45]. In contrast, when ER stress is initiated by glucose deprivation, which leads to a defect in the N-glycosylation important for the proper folding of glycoproteins, each autophagosome carries a single ER fragment [46]. The existence of ER-containing autophagosomes is supported by the juxtaposition of Atg8 and the ER marker protein Sec61 in fluorescence microscopy analyses in yeast [45]. Additionally, *in vitro* and *in vivo* studies in mammals on the Z mutant form of *α*_1_-antitrypsin (*α*_1_-ATZ), which aggregates and accumulates in the ER [47], have shown that the cytoplasmic *α*_1_-ATZ aggregates colocalize with GFP-LC3 and ER resident KDEL-containing proteins [48]. Ultrastructural analyses have confirmed that these structures are indeed ER-containing autophagosomes [36].

Several evidences suggest that the sequestration of ER portions by autophagosomes might be a selective process. In yeast, induction of reticulophagy by DTT results in autophagosomes that contain tightly packed ER fragments that are devoid of cytoplasm [45]. Importantly, immuno-electron microscopy analysis in these cells using anti-GFP antibodies directed against GFP-HDEL, an ER marker protein, has demonstrated that the density of the gold particles is higher inside autophagosomes than in the total cell area [46]. This result is in agreement with the concept of a selective type of autophagy, since in a non-selective scenario the label would have been equally distributed outside and inside of the sequestering vesicles [49]. It cannot be excluded, however, that the increased density of the gold particles is the result of a longer half-life of ER components in the interior of autophagosomes. Further support for a selective nature of this pathway emerges from the notion that the actin cytoskeleton and the selectivity adaptor proteins Atg11 and Atg19 are required for the progression of reticulophagy in yeast (see below).

## 9. Models for the Selective Sorting of ER into Autophagosomes

How is ER targeted for degradation specifically sequestered into autophagosomes? One possibility is that fragments of ER containing unfolded proteins or aggregates are pinching off from the main ER body and are directly transported to the site where autophagosomes arise (Figure 1(b)). During the yeast Cvt pathway, for example, the selective sorting of the cargo oligomer requires the receptor Atg19, the adaptor protein Atg11, and the actin cytoskeleton. Interestingly, these three components have been linked to ER degradation under both stress conditions and nutrient deprivation in yeast [45, 46, 50]. A second possibility is that the selection and enwrapping by autophagosomes occurs in very close proximity to the ER (Figure 1(c)). In contrast to the previous model, this situation does not require a specific machinery to direct the cargo, but rather a system to recruit the Atg proteins to the location where the cargo resides. In both scenarios it remains a mystery how the ER fragments are generated, which factors regulate this scission, and how the ER is selectively sequestered. Interestingly, a recent study in *S. cerevisiae* has shown that Atg8 and Cvt pathway components are recruited onto the ER and negatively regulate the extraction and proteasomal degradation of the misfolded Hmg2 transmembrane protein [51]. Cells could potentially exploit a similar mechanism to recruit the Atg proteins to the ER during reticulophagy. At the ER, the Atg machinery could catalyze the expansion of new membranes destined to sequester an ER fragment or alternatively rearrange a preexisting ER cisterna to constitute the limiting membrane of the sequestering autophagosome. The latter possibility is supported by studies in yeast showing the presence of autophagosomes with ribosomes attached to the membrane surface [45]. In addition, electron tomography analyses in mammalian cells have shown that autophagosomes can be physically connected to the ER, suggesting that these carriers might directly emerge from the ER [52, 53].

## 10. Regulation of Reticulophagy by ER Quality Control Signaling

**Figure 2:**
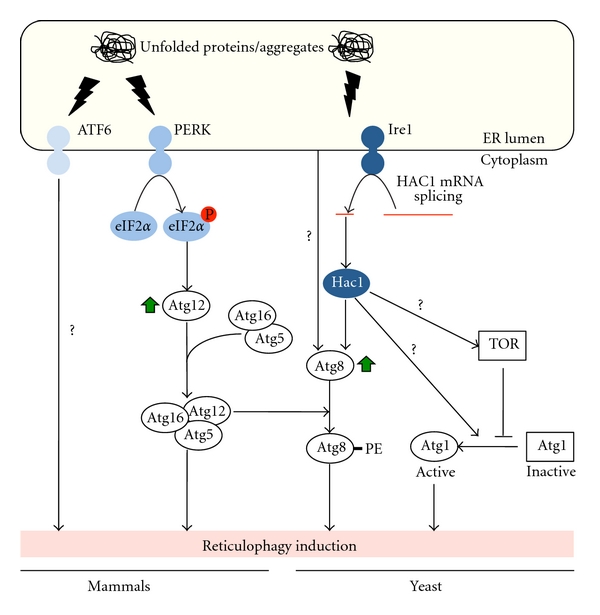
Signalling cascades inducing reticulophagy upon ER stress. The transmembrane protein Ire1 (yeast and mammals), ATF6, and PERK (mammals) sense the accumulation of unfolded proteins and/or aggregates, and trigger a general transcriptional response that affect the levels of proteins involved in autophagy. These include Atg8 (signal mediated through Ire1/Hac1 and unidentified alternative pathways in yeast) and Atg12 (mediated by the PERK/eIF2*α* signalling cascade in mammals). The Atg12-Atg5 (Atg16) complex facilitates the lipidation of Atg8 and autophagy induction. Unknown signalling events in yeast, dependent or independent of the inhibition of the Tor kinase, promote Atg1 activation. Green arrows indicate an increase in protein levels. Question marks indicate signalling cascades that may exist but have not yet been characterized.

The yeast UPR consists of a main signaling pathway initiated by the ER transmembrane kinase inositol requiring enzyme 1 (Ire1). The luminal domain of Ire1 senses the accumulation of unfolded proteins, while the cytoplasmic extension transduces the signal into the nucleus initiating a cellular response at the transcriptional level [54]. Activated Ire1 initiates the nonconventional splicing of *HAC1* mRNA, leading to the production of the transcription factor Hac1, which in turn upregulates the expression of UPR target genes (Figure 2) [54]. Together with the Ire1 counterparts, mammals have two additional ER-stress sensors to induce the UPR: the RNA-dependent protein kinase-like ER kinase (PERK) and the activating transcription factor 6 (ATF6) (Figure 2) [54].

Increasing Atg8 protein levels upon ER stress has been shown to depend on functional Hac1 in yeast (Figure 2) [45]. Additional signaling cascades, however, might be involved in triggering reticulophagy, as the expression of constitutively active spliced Hac1 is not sufficient to stimulate the formation of autophagosomes [45]. Accordingly, cells lacking Hac1 or Ire1 remain capable of inducing the transcription of* ATG8. *This suggests that redundancies or crosstalk among the signaling events regulating autophagy in response to ER stress exist [45].

The lipidation of Atg8/LC3-I also depends on the formation of a large protein complex composed of Atg16 and the conjugate Atg12-Atg5, which is thought to act as an E3-like enzyme conjugating Atg8/LC3-I to PE on autophagosomal membranes [55, 56]. Upregulation of *ATG12*, and the concomitant conversion of Atg8/LC3-I into Atg8-PE/LC3-II, relies on the phosphorylated eIF2*α*, which itself depends on PERK activation after ER stress in mammalian cells (Figure 2) [37, 44].

Atg1/ULK kinase activity is required to coordinate the action of the Atg proteins during the early events of autophagosome biogenesis [7]. Numerous signaling cascades regulating autophagy such as the mTOR, the AMPK, and the PKA pathways modulate the Atg1/ULK function [7]. Interestingly, Atg1 kinase activity is also enhanced upon ER stress in yeast (Figure 2) [33]. It remains to be established how ER stress acts on this kinase, whether through the above-mentioned cascades or via alternative signaling pathways. For example, depletion of sphingosine-1-phosphate (S1P) phosphatases in mammalian cells leads to an increase of endogenous S1P levels, which cause an ER stress that triggers autophagy [57]. This induction is mTor-independent and PERK-, Ire1-, and ATF6-dependent. Moreover, ER stress causes a release of Ca^2+^ from the ER into the cytosol initiating various signaling cascades, some of which are likely to be involved in autophagy induction [58, 59]. While future research is required to understand the signaling networks regulating autophagy in response to ER stress, it is conceivable that reticulophagy could be induced differently depending on the type and intensity of the ER stress.

## 11. Putative Physiological Roles ofReticulophagy

Cells subjected to ER stress contain massively expanded ER with increased total length, distance between the lipid bilayers limiting the cisternae and membrane continuity [45]. These morphological changes are not likely caused by the accumulation of unfolded proteins but rather serve as an adaptive response in order to efficiently buffer the ER stress. This might serve to reduce the concentration of unfolded proteins by increasing the space dedicated to protein folding. This idea is supported by the observation that either yeast expressing the constitutively active Hac1, or mammalian cells with the ectopic expression of its metazoan orthologue Xbp1, two proteins capable of inducing a UPR in the absence of unfolded proteins, exhibit an expanded ER [45, 60]. In addition, mammalian cells in which autophagy has been inhibited or genetically ablated display an extended ER upon stress [43]. Conversely, yeast cells accumulating ER-containing autophagosomes do not contain expanded ER [45]. Together, these observations suggest that autophagy could be important to maintain ER homeostasis during UPR by segregating and/or degrading part of the ER. Thus reticulophagy, through the selective turnover of aggregate-containing and/or damaged ER fragments, could operate in parallel to the ERAD system. This may provide an additional mechanism to dispose unfolded proteins and a way to eliminate damaged membranes. This putative role has been evidenced in yeast expressing pathological mutant versions of human proteins such as the fibrinogen Aguadilla mutation and *α*_1_-ATZ, which accumulate as unfolded aggregates in the ER [34–36]. Knockout strains lacking *ATG* genes expressing these pathological proteins more rapidly amass large amounts of protein aggregates compared to wild-type cells. This suggests that autophagy is important during conditions where the ERAD system is overwhelmed [34, 35]. A similar phenotype was observed in mouse cells lacking Atg5 and expressing expanded polyglutamine repeats [44, 61]. These proteins form cytoplasmic aggregates that trigger ER stress, possibly by impairing ERAD and thus causing an accumulation of unfolded proteins in the ER. Therefore, basal autophagy could serve a similar protective role by preventing the accumulation of misfolded proteins in nonstressed cells. This idea is supported by the observation that an autophagy block caused by the deletion of *ATG6 *also induces a UPR in non-stressed cells [35]. The direct implication of autophagy as an ER housekeeping pathway, however, needs to be analyzed in more detail as Atg6 is also required for endosomal trafficking [62, 63].

Paradoxically, autophagy displays a double role in cell viability. It is able to increase the lifespan by protecting against cellular damage; however, in specific pathological situations or when cells have undergone irreversible stress or injuries, autophagy can also contribute to cell death [64]. How reticulophagy contributes to cell fate is not clear and current available data are in part contradictory. Ogata and coworkers concluded that autophagy has a protective role against ER stress-induced cell death as autophagy-deficient cells show higher vulnerability to ER stress and conversely, pretreatment with rapamycin makes cells more resistant to this damage [65]. In contrast, Ding and collaborators proposed a dual role for autophagy according to the status of the cells; autophagy promotes cell survival in cancer cells displaying ER stress, and induces cell death in nononcogenic cells [43]. In yeast, an intact autophagy machinery is essential for cell growth under strong UPR-inducing conditions [45]. Interestingly, it has been proposed that the engulfment of the ER by autophagosomes, without the degradation of the sequestered cargo, is sufficient for autophagy to mitigate ER stress [45]. This hypothesis has been underscored by the finding that in the presence of high concentrations of tunicamycin, an inhibitor of protein glycosylation, Atg proteins are necessary for cell survival while vacuolar proteases are dispensable [45]. Under the same circumstances, ER-containing autophagosomes do not fuse with vacuoles when ER stress is maintained for longer periods [45]. In contrast, when ER stress is initiated by glucose depletion, ER fragments are transported to the lumen of the vacuole indicating that a complete autophagy process occurs [46]. Additional studies are necessary to understand the exact contribution of autophagy as an ER stress response mechanism. A possible scenario is that reticulophagy could have been adapted to differentially modulate its response according to the nature of the stress, and the status of the cell and/or the tissue.

## 12. Conclusions

Despite their potential relevance in physiological and pathological contexts, the regulation and mechanisms of ribophagy and reticulophagy remain largely unknown. It remains to be determined which of the known or if novel Atg proteins mediate the recognition and selective sequestration of ribosomes and ER fragments into autophagosomes. Moreover, how the cell regulates the segregation of the unwanted parts of the ER and how this breaks away from the organelle need to be further analyzed. A vast field is waiting to be explored.
